# Changes in Cortical Plasticity in Relation to a History of Concussion during Adolescence

**DOI:** 10.3389/fnhum.2017.00005

**Published:** 2017-01-17

**Authors:** Sean K. Meehan, Jasmine L. Mirdamadi, Douglas N. Martini, Steven P. Broglio

**Affiliations:** School of Kinesiology, University of MichiganAnn Arbor, MI, USA

**Keywords:** concussion, mild traumatic brain injury, plasticity, neurophysiology, transcranial magnetic stimulation, intracortical facilitation, short-intracortical inhibition

## Abstract

Adolescence and early adulthood is a critical period for neurophysiological development potentially characterized by an increased susceptibility to the long-term effects of traumatic brain injury. The current study investigated differences in motor cortical physiology and neuroplastic potential across a cohort of young adults with adolescent concussion history and those without. Transcranial magnetic stimulation (TMS) was used to assess motor evoked potential (MEP) amplitude, short-interval cortical inhibition (SICI) and intracortical facilitation (ICF) before and after intermittent theta burst stimulation (iTBS). Pre-iTBS, MEP amplitude, but not SICI or ICF, was greater in the concussion history group. Post-iTBS, the expected increase in MEP amplitude and ICF was tempered in the concussion history group. Change in SICI was variable within the concussion history group. *Post hoc* assessment revealed that SICI was significantly lower in individuals whose concussion was not diagnosed at the time of injury compared to both those without a concussion history or whose concussion was medically diagnosed. Concussive impacts during adolescence appear to result in a persistent reduction of the ability to modulate facilitatory motor networks. Failure to report/identify concussive impacts close to injury during adolescence also appears to produce persistent change in inhibitory networks. These findings highlight the potential long-term impact of adolescent concussion upon motor cortical physiology.

## Introduction

Mild traumatic brain injury or concussion, has traditionally been associated with transient declines in cognitive and motor function. In most cases neuropsychological functioning resolves to pre-injury benchmarks within 7–10 days of the injury (McCrory et al., 2013). This timeline parallels the acute neurometabolic events triggered by the concussive impact (Giza and Hovda, 2014) propagating the belief that concussive effects are functional in nature and brief. However, the transient nature of concussion has come under increasing scrutiny with reports linking the injury to long-term declines in cognitive functioning (Moser and Schatz, 2002; De Beaumont et al., 2009), depression (Guskiewicz et al., 2007; Kerr et al., 2012) and mild cognitive impairment (Guskiewicz et al., 2005).

To better define the speculative link between long-term cognitive health and concussion (Castellani et al., 2015; Iverson et al., 2015; Maroon et al., 2015) several controlled studies to investigate persistent effects of concussion beyond the acute phase have been conducted. Of particular interest are persistent changes in asymptomatic individuals that foreshadow clinically relevant declines in cognitive and motor function with age (De Beaumont et al., 2009; Tremblay et al., 2013). The predictive nature of neuropsychological testing with respect to concussion history declines as time from injury increases (Broglio et al., 2006, 2007). Likewise questionable sensitivity of anatomical assessments like computerized tomography and structural magnetic resonance imaging in the acute phase of concussion raises concerns about their sensitivity to persistent alterations in anatomy (Jordan and Zimmerman, 1990). Diffusion based assessments of white matter integrity demonstrate greater sensitivity to the persistent effects of the shearing and stretching of axons generated by the rapid acceleration and deceleration of the brain at time of injury. For example, increased fractional anisotropy and reduced mean diffusivity across the whole brain in adolescents 2 months post-injury highlights persistent decreases in white matter integrity despite no significant difference in clinical testing (Virji-Babul et al., 2013). In collegiate football players, changes in functional anisotropy seen in the corpus callosum and cortical spinal tracts days after concussion are still present at 6 months post-injury (Henry et al., 2011). More worrisome, decreased fractional anisotropy and increased mean diffusivity in the corpus callosum, right inferior and bilateral superior longitudinal fasciculus in older retired collegiate athletes correlate with the extent of cognitive decline (Tremblay et al., 2014b).

Much of the evidence for persistent neurophysiological change in asymptomatic individuals months and years after the concussive injury comes from electrophysiological markers. Asymptomatic young adults with a history of concussion in adolescence demonstrate persistent reductions in psychophysiological indices such as the P3b and N2 event related brain potentials (Broglio et al., 2009; Moore et al., 2014) indicative of subclinical alterations in attention allocation (Donchin and Coles, 1988) and response inhibition during sensorimotor conflict (Larson et al., 2014). The absence of overt functional deficits (Broglio et al., 2009), despite altered electrophysiology, has led to speculation that clinically relevant behavioral declines may only occur with a decreasing brain/cognitive reserve with age that reduces an individual’s ability to compensate for the neurophysiological alterations post-concussion (Broglio et al., 2012). Such a theory is appealing as differing rates of diminishing reserve may account for variability in the chronic consequences of concussion. To this effect, attenuation of the P3b and P3a event-related potential components in retired athletes correlates with compromised declines in episodic memory and response inhibition (De Beaumont et al., 2009).

The impact of concussion upon the motor system is evidenced by acute hyperactivation of both dorsolateral prefrontal and parietal cortex during voluntary sequential movement (Jantzen et al., 2004), persistent reductions in levels of implicit learning (De Beaumont et al., 2012) and long-term impairment in coordination, ataxia and spasticity (Rabadi and Jordan, 2001). Such motor symptoms may result from direct injury to the motor cortex or from altered connectivity between prefrontal cortex and motor cortical areas. Regardless of etiology, assessments of motor cortical physiology may provide another pathway by which to evaluate the potential chronic effects of concussion. Static group comparisons of transcranial magnetic stimulation (TMS) derived measures of motor cortex excitability across concussed and non-concussed cohorts within both the acute and chronic phases post-injury have produced variable results (for reviews, see Lefebvre et al., 2015; Major et al., 2015). Resting motor threshold (RMT), an index of membrane excitability, in the acute and chronic phases of concussion is generally consistent with that of non-concussed controls, however, two studies have reported elevated RMT 2-weeks post-injury that normalized by 3-months post-injury (Chistyakov et al., 1998, 2001) while two others demonstrate persistent elevations up to 5 years post-injury (Tallus et al., 2012, 2013). Intracortical facilitation (ICF), mediated by a mix of glutamatergic and gamma-aminobutyric acid (GABA)-ergic mechanisms, was reduced at 1 and 4 weeks post-injury in one study (Powers et al., 2014) but enhanced over a similar time period in another study (Bashir et al., 2012). Regardless of direction, other studies suggest that any acute changes in ICF normalize in asymptomatic individuals (De Beaumont et al., 2007, 2009). Static comparisons of the inhibitory marker short-interval intracortical inhibition (SICI), a GABA_A_ mediated form of inhibition, have largely failed to differentiate between concussion and control groups at the acute (Bashir et al., 2012; Powers et al., 2014; Pearce et al., 2015) and chronic (De Beaumont et al., 2007, 2009; Tremblay et al., 2011) stages post-injury, however, there has been a report of reduced SICI in retired Australian football players (Pearce et al., 2014). Of all the TMS measures of motor cortex excitability, the most consistent finding in concussed individuals is an enhanced cortical silent period duration, a GABA_B_ mediated form of intracortical inhibition (Tremblay et al., 2011; De Beaumont et al., 2012; Miller et al., 2014; Pearce et al., 2014, 2015) that appears to persist (De Beaumont et al., 2007) or is even enhanced (De Beaumont et al., 2009) as time from injury increases. However, even though cortical silent period is enhanced in younger active Australian football players with a history of concussion (Pearce et al., 2015) in a cohort of older retired players the silent period was decreased (Pearce et al., 2014). Interestingly, the cortical silent period is the only measure mentioned above that requires active engagement by the participant given the requirement to monitor and maintain a fixed level of volitional contraction. In contrast to static comparisons of motor cortex excitability, relatively few studies have investigated the neural plastic potential of these measures in symptomatic (Chistyakov et al., 1998; Bashir et al., 2012; Tremblay et al., 2015) and asymptomatic individuals (De Beaumont et al., 2012; Tremblay et al., 2015). These studies have all focused upon changes in RMT, motor evoked potential (MEP) amplitude and cortical silent period. In a series of case studies, Tremblay et al. (2015) demonstrated that continuous theta burst stimulation, a patterned TMS protocol that typically decreases MEP amplitude, had no such effect 2-weeks post-concussion but that the expected MEP reduction had returned by 6-weeks. In a cohort of retired collegiate athletes, De Beaumont et al. (2012) demonstrated reduced efficacy of both facilitatory and inhibitory paired-associative stimulation protocols, a plasticity inducing protocol that pairs trains of electrical median nerve and motor cortical TMS, to modulate MEP amplitude and cortical silent period duration in those with a history of concussion. However, none have assessed the persistent impact of adolescent concussion upon neuroplastic potential and none have assessed changes in the ability to facilitate the intracortical networks mediating SICI and ICF.

Both iontropic GABA_A_ and metabotropic GABA_B_ mediated mechanisms are associated with motor performance, learning and neural plasticity (Davies et al., 1991; Coxon et al., 2014). Application of the GABA_B_ agonist baclofen suppresses long-term potentiation (LTP) like plasticity indicating a potential consequence of chronic enhancements in GABA_B_ mediated inhibition is reduced potential for LTP. This relationship was demonstrated in the paired associative stimulation study performed by De Beaumont et al. (2012) where enhanced GABA_B_-mediated intracortical inhibition was associated with decreased effectiveness of the paired associative stimulation protocol and implicit motor learning. Disinhibition in the form of reduced GABA_A_ mediated inhibition, as indexed by SICI, also appears to be an important indicator of motor performance (Heise et al., 2013), learning (Coxon et al., 2014) and plasticity (Murase et al., 2015). However, excessive reductions in resting and event-related SICI may also become maladaptive contributing to loss of muscle differentiation (Bernard and Seidler, 2012) and poorer manual dexterity (Heise et al., 2013) seen in older adults. Whether the ability to modulate GABA_A_ mediated inhibition, not typically captured in the static group assessments where the targeted muscle is at rest, is compromised in individuals with a history of concussion during adolescence and young adulthood, periods of heightened plasticity (Toledo et al., 2012; Fuhrmann et al., 2015) is unknown. Any persistent reduction in GABA_A_ levels associated with use-dependent plasticity prior to reaching full maturation in early adulthood may be problematic by leading to too much disinhibition and exacerbate loss of muscle differentiation that occurs with natural aging (Bernard and Seidler, 2012).

The purpose of the current study was to investigate the persistent changes in motor cortical plasticity in asymptomatic young adults who last suffered a concussion during adolescence. Given the relationship between concussion, GABA_B_ function and LTP seen in collegiate athletes who have suffered concussion, we hypothesized that individuals who sustained a concussion during their teen years would demonstrate reductions in N-methyl-D-aspartate (NMDA) receptor mediated LTP-like plasticity even in the absence of baseline differences in motor cortical physiology and behavior. We used the NMDA-receptor dependent variant of repetitive TMS known as intermittent theta burst stimulation (iTBS) to induce transient motor cortical plasticity (Huang et al., 2005, 2007).

## Materials and Methods

### Participants

Thirty-one self-reported right-handed adults (20 males, 11 females, 21 ± 2.4 years) were recruited from the general student body of the University of Michigan. All participants provided written informed consent; the Institutional Review Board of the University of Michigan Medical School (IRBMED) approved the study protocol. No vulnerable populations were involved.

Participants were initially categorized in to one of two groups based upon self-report of concussion. The self-report involved two mutually exclusive questions: (1) “Have you ever been told by a medical professional (e.g., doctor, athletic trainer, EMT, nurse) that you had a concussion?”; and (2) “Following a blow to the head, have you ever experienced any of the following symptoms?”. The second question was followed by a list of common concussion symptoms used in clinical diagnostic interviews (McCrory et al., 2013). The list of symptoms included headache, difficulty concentrating or focusing, feeling slowed down, dizziness of balance problems, nausea, fatigue/lack of energy, feeling like you are in a fog, irritable, drowsiness, memory loss surrounding injury, sensitivity to light, loss of balance, sensitivity to noise and blurred vision. Individuals were asked to list each discrete event that fell under each question. Individuals were asked to provide their age at the time of event, the approximate month and year in which the event occurred, the sport being played at time of event and estimated duration of symptoms. Detailed information about injury management at the time of the event and time to return to play were not included in the concussion questionnaire. Participants were instructed that the same event could not be listed under both questions. Individuals who answered *no* to both questions were placed in the no concussion history group. Individuals who answered *yes* to at least one of the two questions were placed in the concussion history group. Based upon these criterion 16 individuals were assigned to the concussion history group and 15 individuals were assigned to the no history group (Table 1). *Post hoc*, the concussion history group was further subdivided into a “diagnosed” concussion group and a group who reported a “concussion-like” event that was not formally diagnosed by a medical professional. Individuals were assigned to the diagnosed group if they answered yes to the first question about medical diagnosis (*n* = 9). Individuals were assigned to the concussion-like group if they answered no to the first question but answered yes to the second question regarding the list of common symptoms (*n* = 7). Two of the nine individuals in the diagnosed group answered yes to both the first and second questions. In both cases these participants identified separate events under each question, with one event being medically diagnosed and a separate concussion-like event that they did not report. In both cases the chronological order of events involved the first concussive injury being medically diagnosed (reported under Question 1).

**Table 1:** **Demographic data for No History and Concussion groups**.

Variable	Group			
	No History	Concussion	Diagnosed^1^	Concussion-like History^1^
Sample size	15	16	9	7
Age (in years)	21 ± 2.5	20 ± 2.3	21 ± 2.7	19 ± 1.2
Sex	8M, 7F	12M, 4F	7M, 2F	5M, 2F
Body mass index	24 ± 5	25 ± 5	24 ± 4	28 ± 7
Education (years)	15 ± 1.4	14.6 ± 2.2	15 ± 2.4	13 ± 1.1
Number of concussions	–	2 ± 1.2	2 ± 1.5	2 ± 1.1
Age of last injury (years)	–	16 ± 1.6	16 ± 1.4	16 ± 2
Time since last injury (years)	–	4 ± 3	5 ± 3	3 ± 2
Loss of consciousness	–	6	5	1
Amnesia	–	7	6	1
Duration of symptoms (days)	–	4 ± 3.6	5 ± 4.11	3 ± 2.9
Years playing sports—high school^2,3^	3 ± 1.5	4 ± 0.9	3.3 ± 1.1	3.8 ± 0.4
Years playing sports—middle school^4,5^	2 ± 1.4	3 ± 0.5	3 ± 0.5	3 ± 0.5

*Where appropriate data are reported as mean ± standard deviation. 1. These subgroups are derived from the Concussion History group. 2. No History group reported playing: No Sport (3), Running (2), Tennis (2), Swimming (2), Baseball/Softball (2), Basketball (1), Soccer (1), Figure Skating (1), Wrestling (1). 3. Concussion History group reported playing: Soccer (6), Football (4), Baseball (2), Track (2), Volleyball (1), Wresting (1). 4. No History group reported playing: No Sport (3), Basketball (3), Running (3), Swimming (2), Baseball/Softball (1), Soccer (1), Figure Skating (1), Skiing (1). 5. Concussion History group reported playing: Soccer (7), Football (5), Basketball (2), Baseball (1), Gymnastics (1)*.

Exclusion criterion included individuals with a diagnosed concussion or self-reported concussion-like symptoms after the age of 18 and/or individuals being treated for post-concussion symptoms at the time of screening. Individuals with a history of a developmental or learning disorder, attention deficit disorder, neuropsychiatric disorder, epilepsy, brain surgery, alcohol of drug abuse, use of psychotropic medications or any other contraindication to TMS (Keel et al., 2001) were also excluded. Exclusion criterion were assessed from the self-reported answers collected as part of the health-history, concussion-history and TMS screening questionnaires.

### Experimental Design and Procedure

**Figure 1:**
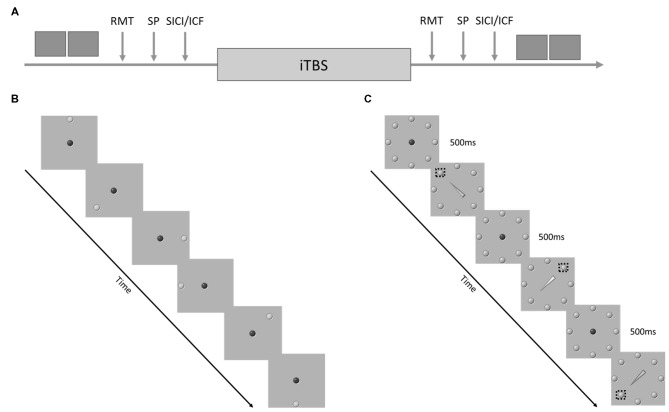
**(A)** Experimental Procedure. The dark gray boxes represent behavioral task performance. RMT, resting motor threshold; SP, single pulses delivered at 120% of RMT, short-interval cortical inhibition (SICI)/intracortical facilitation (ICF) = paired pulse stimulation with an inter-pulse interval of either 2 ms or 15 ms. **(B)** Example of the sequence of events during the simple response task. **(C)** Example of the sequence of events during the cued response task. The dashed boxes represent the correct target for each cue.

All participants completed a single testing session (Figure 1A). Following TMS screening, participants completed health-history and concussion-history questionnaires. Upon completion of the questionnaires participants completed the simple and cued response tasks (~5 min). The order of behavioral tasks was randomized across participants. Participants then moved to the TMS testing chair where electromyography electrodes were placed over the right first dorsal interosseous (FDI) and the participant was co-registered to the BrainSight^TM^ stereotactic guidance system (Rogue Research, Montreal, QC, Canada; ~5 min). Motor cortical hotspot, RMT, active motor threshold and test stimulus intensity were determined (~15–20 min). Following a 2–3 min delay for set-up MEP amplitude, SICI and ICF were assessed (~10 min). Order of TMS assessments was randomized across participants. Upon completion of the baseline testing a 2–3 min delay followed before administration of iTBS. There was at least a 5–8 min delay between the end of the iTBS and the start of the post-iTBS physiological/behavioral assessments. Following iTBS thresholds and test stimulus intensity were redefined to control for shifts in cortical excitability. Pre-iTBS stimulus intensity was used for the post-iTBS assessment of MEP amplitude to remain sensitive to changes in cortical excitability. Finally, EMG electrodes and the BrainSight^TM^ subject tracker hardware were removed before participants completed the simple and cued response tasks.

### Response Tasks

Two targeted reaching tasks were used to assess motor performance: the simple response task and the cued response task. These tasks were chosen as they index similar domains of attention and response inhibition evaluated in common clinical assessments of concussion.

The order of the tasks was randomized. Both tasks required participants to perform discrete reaching movements from a center “home” target to a target that appeared at one of eight equidistant radial positions (Figure 1B). Participants stood in front of a 46″ touch screen (PS4660T, Planar Systems Inc., Beaverton, OR, USA) at a distance that allowed them to reach comfortably to all target positions. Target presentation and data acquisition was conducted using a variation of custom software (LabVIEW 2014, National Instruments, Austin, TX, USA; Meehan et al., 2011; Brodie et al., 2014).

The simple response task started with the appearance of a red home target located in the center of the touch screen. Participants were required to move to the home target (50 mm diameter) and hold contact with the screen for 500 ms to trigger the start of the trial. A green target (50 mm diameter) then appeared at one of the eight equidistant positions. The distance between the center of the red home target and the green target was 200 mm. Participants were instructed to reach and touch the center of the green target as fast and accurately as possible by taking the most direct route. Once the participant touched the screen the green target disappeared and participants were instructed to return to the home target at their own pace. Fifty reaching trials were completed before and after iTBS. Reaction time was defined as the time between green target appearance and lift-off from the home target. Movement time was defined as the time between lift off from the home target and touchdown inside the green target. Error trials were classified as any touch on the screen that did not fall inside the green target. Error trials were not included in the derivation of reaction time and movement time.

The cued response task proceeded similarly to the simple response task. The only differences were: (1) all eight radially arrayed targets were presented statically on the screen; and (2) the target to reach toward was determined by a central arrow cue that replaced the red home target. If the arrow was green, participants were instructed to reach to the target at the point of the arrow. If the arrow was yellow participants were instructed to reach to the target opposite the tip (Figure 1C). All other aspects of task presentation and timing were identical to the simple response task. Fifty-trials of the cued response task were completed pre- and post-iTBS. Within each set of 50 trials 70% of the cues were valid, 30% of the cues were invalid.

For the cued response task, reaction time was defined as the time between arrow cue appearance and lift-off from the home target. Movement time was defined as the time between lift off from the home target and touchdown on the screen. Error trials were classified as any touch on the screen that did not fall inside the correct target.

### Transcranial Magnetic Stimulation (TMS) and Recording

TMS was delivered using a MagVenture MagPro X100 with option stimulator (MagVenture Inc., Atlanta, GA, USA) and a statically cooled figure-8 coil (MCF-B70). The coil was placed over the optimal scalp position to elicit MEPs in the FDI. The coil was oriented tangentially with the handle 45° to the midline in a posterior lateral orientation. The location and trajectory of the FDI motor cortical hotspot was marked using the BrainSight^TM^ stereotactic system.

MEPs in the FDI were recorded using surface electromyography (PowerLab 8/30, AD Instruments, Colorado Springs, CO, USA). Surface electromyography recording was triggered from the TMS stimulator using a 5V TTL pulse with an epoch of −0.3 to 0.5 s. During acquisition, data were amplified (×1000), digitized (×5000 Hz) and filtered (band pass filtered 5–1000 Hz, notch filter −60 Hz).

Cortical excitability was assessed pre and post-iTBS using sixteen monophasic single pulse (SP) TMS stimuli delivered at 120% of pre-iTBS RMT. TMS stimuli were monophasic with a posterior-anterior current direction. RMT was defined as the percentage of stimulator output that elicited an MEP of ≥50 μV peak to peak on 5 out of 10 trials in the targeted muscle. MEP amplitude was defined as peak-to-peak amplitude.

SICI and ICF were assessed by preceding a supra-threshold TMS stimulus with a sub-threshold conditioning stimulus (Kujirai et al., 1993). Both the conditioning and suprathreshold TMS stimuli were monophasic with a posterior-anterior current direction. The intensity of the sub-threshold conditioning stimulus was set to 80% of active motor threshold. Active motor threshold was defined as the percentage of stimulator output that elicited an MEP of ≥200 μV peak to peak on 5 out of 10 trials during FDI contraction at 20% of the maximum force production. The intensity of the suprathreshold test stimulus was set to the stimulator output that produced an MEP of 1 mV peak-to-peak amplitude (Kujirai et al., 1993). The intensity of the conditioning and suprathreshold test stimulus was redefined following iTBS. For SICI 16 trials were conducted using an inter-stimulus interval of 2 ms. For ICF 16 trials were conducted with an interval of 15 ms. Sixteen unconditioned trials were included in which only the test stimulus was delivered. SICI and ICF were defined as the peak-to-peak MEP amplitude for conditioned trials divided by the peak-to-peak MEP amplitude. Values were expressed as a percentage. Values below 100% reflect inhibition relative to the unconditioned MEP amplitude. Values greater than 100% represent facilitation relative to the unconditioned MEP amplitude.

iTBS consisted of three biphasic TMS stimuli (AP-PA current direction) presented at 50 Hz, repeated every 200 ms for 2 s at an intensity of 80% of active motor threshold. The 2 s bursts were repeated every 8 s for a total of 600 magnetic stimuli over 190 s (Huang et al., 2005).

### Data Analysis

All statistical analyses were performed using IBM SPSS Statistics version 24 (IBM Corp, Armonk, NY, USA). Differences across the concussion history and no history groups across both the behavioral and physiological dependent variables were assessed using separate Group (No History, History) × Time (Pre-iTBS, Post-iTBS) × Sex (Female, Male) mixed measures analysis of variance (ANOVAs). Group and Sex were treated as between subject factors. Time was treated as a repeated measure. Sex was included as a factor to account for unequal numbers of males and females across the No History and History groups. Significant interactions were decomposed using two methods. First, to establish potential baseline differences between the groups any interaction involving the factor Group was decomposed using a one-way between groups ANOVA to compare the pre-iTBS values between groups. Second, to determine whether iTBS had a significant effect upon a given measure significant interactions involving the effect of Time were also decomposed using linear contrasts to compare pre- and post-iTBS values within each group.

The behavioral dependent variables for the simple response task included reaction time, movement time and accuracy. For the cued task the dependent measure were the reaction time and movement time costs, defined as the difference between validly and invalidly cued trials. Accuracy on validly cued trials was also assessed. Physiological dependent variables included MEP amplitude, SICI, ICF and RMT. Variables that violated the assumption of normality were log transformed prior to analysis. The log transformed variables included: simple and cued response task accuracy and MEP amplitude.

As a secondary *post hoc* analysis, we derived measures of effect size for the Diagnosed and Concussion-Like subgroups based on self-report of concussion in the Health History Questionnaire. Inspection of individual responses of the concussion history group revealed that SICI’s response to iTBS was variable across individuals. The *post hoc* analysis was conducted to determine whether the variable response of SICI to iTBS could be attributed to greater random variation across subjects (Hamada et al., 2013) or whether the concussive injury was diagnosed by a medical professional (*yes* response to question 1 above, *n* = 9) or went unreported/undiagnosed at the time of injury (*yes* response to question 2 above, *n* = 7). Consistent with the primary analyses effect sizes were used to baseline differences and differences in the efficacy of iTBS. Baseline differences were quantified using Cohen’s *d* for each concussion history subgroup (Diagnosed, Concussion-Like) and the No History group. The efficacy of iTBS was assessed using Cohen’s *d* to compare the percentage change following iTBS across the concussion history subgroups and the No History group.

## Results

**Figure 2:**
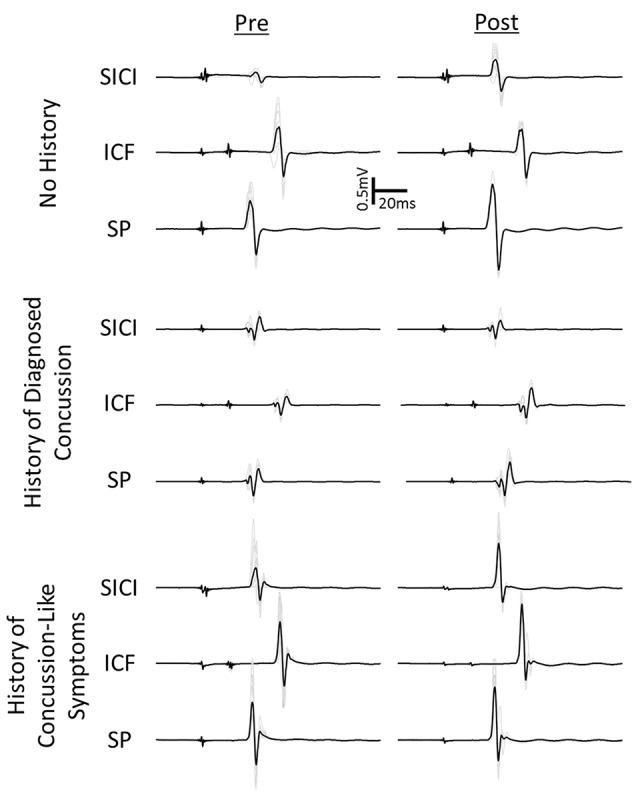
**Example motor evoked potential (MEPs) pre and post-intermittent theta burst stimulation (iTBS) for one participant from the no history and two participants from the concussion history group.** One individual from the concussion history group had a concussion diagnosed by a medical professional (diagnosed). The other individual reported concussion like symptoms that went either unreported or undiagnosed as concussion at the time (concussion-like history).

Table 2 shows the means and standard deviations for the simple and cued response tasks. Figure 2 shows example MEPs from one subject with no concussion history, a history of diagnosed concussion and concussion-like history before and after iTBS.

**Table 2:** **Behavioral data from the simple and cued response tasks (mean ± standard deviation)**.

Variable	Group							
	No History		Concussion		Diagnosed^1^		Concussion-like History^1^	
	Pre	Post	Pre	Post	Pre	Post	Pre	Post
**Simple response task**
Reaction time (ms)	365 ± 42	367 ± 39	368 ± 28	373 ± 34	364 ± 12	366 ± 12	373 ± 14	382 ± 14
Movement time (ms)	299 ± 37	296 ± 34	331 ± 46	312 ± 50	324 ± 14	306 ± 15	340 ± 16	320 ± 17
Accuracy (%)	96 ± 4	96 ± 5	97 ± 2	96 ± 4	98 ± 1	97 ± 1.5	96 ± 1	94 ± 2
**Cued response task—valid**
Response time (ms)	513 ± 16	483 ± 13	520 ± 15	496 ± 13	506 ± 20	482 ± 17	538 ± 23	513 ± 20
Movement time (ms)	362 ± 17	342 ± 18	381 ± 16	362 ± 17	369 ± 22	357 ± 23	397 ± 25	368 ± 25
Accuracy (%)	77 ± 9	77 ± 10	77 ± 8	75 ± 8	77 ± 8	75 ± 8	76 ± 9	75 ± 8
**Cued response task—invalid**
Response time (ms)	534 ± 19	500 ± 19	425 ± 25	395 ± 21	539 ± 24	525 ± 24	561 ± 27	558 ± 28
Movement time (ms)	402 ± 27	392 ± 22	548 ± 18	539 ± 18	425 ± 35	398 ± 28	424 ± 39	391 ± 32

*1. These subgroups are derived from the concussion history group*.

### Simple Response Time task

The Group × Time × Sex ANOVAs for reaction time and log accuracy failed to reveal any significant effects. *Post hoc* effect size calculations comparing the magnitude of the effects in the Diagnosed and Concussion-Like cohorts revealed consistently small effects across each cohort for reaction time (Diagnosed, Cohen’s *d* = 0.04; Concussion-Like, Cohen’s *d* = −0.19) and accuracy (Diagnosed, Cohen’s *d* = 0.04; Concussion-Like, Cohen’s *d* = 0.04). Likewise pre to post-iTBS changes in reaction time and accuracy were relatively small and consistent across the concussion cohorts for reaction time (Diagnosed, Cohen’s *d* = 0.03; Concussion-Like, Cohen’s *d* = 0.28) and accuracy (Diagnosed, Cohen’s *d* = 0.20; Concussion-Like, Cohen’s *d* = 0.32).

The Group × Time × Sex ANOVA for movement time revealed a significant main effect of Time (*F*_(1,27)_ = 7.38, *p* = 0.011, *η*^2^ = 0.13) as well as a strong trend for a significant Group × Time interaction (*F*_(1,27)_ = 4.14, *p* = 0.052, *η*^2^ = 0.22). None of the other interactions or main effects within the Group × Time × Sex model were significant. The main effect of time reflects reduced movement times post-iTBS (303 ± 9 ms) compared to pre-iTBS (313 ± 8 ms) for both groups. However, the strong Group × Time trend was driven by significantly slower movement times pre-iTBS for the Concussion History compared to the No History group (*p* = 0.04) but no difference between the groups post-iTBS (*p* = 0.32).

*Post hoc* effect size calculations comparing the magnitude of the effects pre-iTBS revealed a stronger slowing of movement for the Concussion-Like group (Cohen’s *d* = −1.19) compared to the Diagnosed group (Cohen’s *d* = −0.54). However, both groups demonstrated consistent magnitude of improvement from pre- to post-iTBS (Diagnosed, Cohen’s *d* = 0.82; Concussion-Like, Cohen’s *d* = 0.88).

### Cued Response Time Task

The Group × Time × Sex ANOVAs for reaction time, movement time and log accuracy failed to reveal any significant interactions or main effects. Comparison of effect sizes in the Diagnosed and Concussion-Like cohorts revealed small effects for reaction time (Diagnosed, Cohen’s *d* = 0.40; Concussion-Like, Cohen’s *d* = 0.09), movement time (Diagnosed, Cohen’s *d* = 0.28; Concussion-Like, Cohen’s *d* = −0.29) and log accuracy (Diagnosed, Cohen’s *d* = 0.02; Concussion-Like, Cohen’s *d* = 0.15) pre-iTBS. Both groups also demonstrated consistent magnitudes of improvement post-iTBS in reaction time (Diagnosed, Cohen’s *d* = 0.85; Concussion-Like, Cohen’s *d* = 1.25), movement time (Diagnosed, Cohen’s *d* = 0.01; Concussion-Like, Cohen’s *d* = 0.06) and accuracy (Diagnosed, Cohen’s *d* = 0.01; Concussion-Like, Cohen’s *d* = 0.19).

### Motor Thresholds

Active motor threshold, used to set iTBS stimulation intensity, was 34 ± 2% and 34 ± 2% of stimulator output for the No History and History groups respectively. A Group × Sex between group ANOVA failed to reveal any significant effects upon active motor threshold.

RMTs pre-iTBS were 42 ± 2% and 41 ± 2% of stimulator output for the No History and History groups respectively. RMTs post-iTBS were 42 ± 2% and 41 ± 2% of stimulator output. The Group × Time × Sex mixed measure ANOVA for RMT failed to reveal any significant interaction or main effects.

### Cortical Spinal Excitability

**Figure 3:**
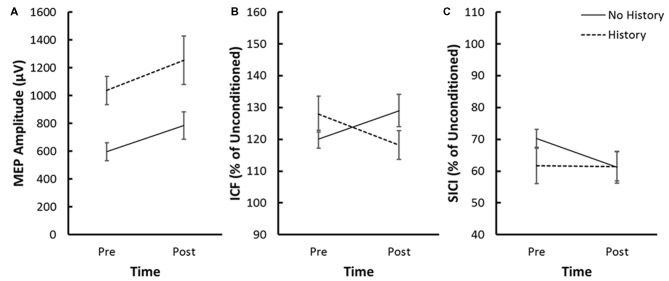
**(A)** Average MEP amplitude, **(B)** average ICF and** (C)** average SICI pre and post-iTBS for the no history and concussion history groups. ICF and SICI are expressed as a percentage of unconditioned MEP amplitude. Error bars represent the standard error of the mean.

The Group × Time × Sex mixed measure ANOVA for the log of MEP amplitude revealed a significant Group × Time interaction (*F*_(1,27)_ = 4.68, *p* = 0.04, *η*^2^ = 0.15; Figure 3A). Decomposition comparing pre-iTBS MEP amplitude across Group revealed that pre-iTBS MEP amplitude was significantly greater for the History compared to No History group (*F*_(1,29)_ = 16.15, *p* = 0.0004, *η*^2^ = 0.15). Linear contrasts comparing MEP amplitude across Time within each Group revealed that the No History group demonstrated a significant increase in MEP amplitude following iTBS (*p* = 0.01) that was not seen in the History group (*p* = 0.95). None of the other interactions or main effects within the Group × Time × Sex ANOVA model were significant.

The magnitude of the enhanced MEP amplitudes prior to iTBS was similar across the Diagnosed (Cohen’s *d* = 1.78) and Concussion-Like (Cohen’s *d* = 1.30) cohorts defined by our health history questionnaire. Likewise the magnitude of the reduction in iTBS efficacy was also consistent across the Diagnosed (Cohen’s *d* = −0.70) and Concussion-Like (Cohen’s *d* = −0.61) cohorts.

### Intracortical Facilitation (ICF)

The Group × Time × Sex mixed measure ANOVA for ICF revealed a significant Group × Time interaction (*F*_(1,27)_ = 6.80, *p* = 0.015, *η*^2^ = 0.20; Figure 3B). Decomposition comparing ICF across the History and No History groups failed to reveal a significant difference in ICF pre-iTBS (*F*_(1,29)_ = 1.19, *p* = 0.23, *η*^2^ = 0.05). Linear contrasts comparing ICF across Time within each Group revealed a significant increase in ICF in the No History group (*p* = 0.043) but a slight but non-significant reduction in ICF for the History group (*p* = 0.15). None of the other interactions or main effects within the Group × Time × Sex ANOVA model were significant.

Effect size calculations revealed that both the Diagnosed (Cohen’s *d* = 0.43) and Concussion-Like (Cohen’s *d* = 0.56) cohorts consistently demonstrated marginally greater ICF pre-iTBS. Likewise the significant reduction in the efficacy of iTBS upon ICF was consistent across concussion history subgroup (Diagnosed, Cohen’s *d* = −0.72; Concussion-Like, Cohen’s *d* = −0.83).

### Short-Interval Cortical Inhibition (SICI)

**Figure 4:**
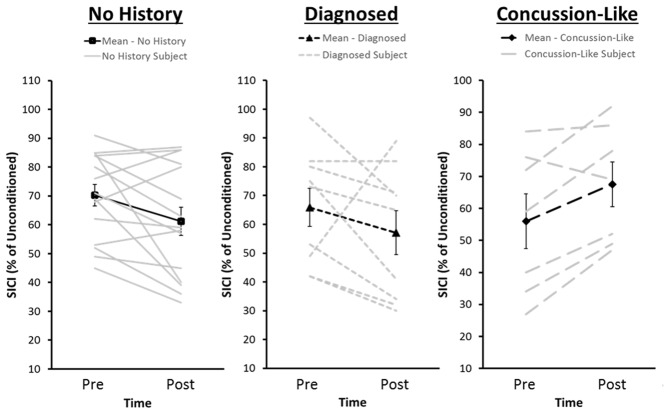
**Distribution of pre- and post-iTBS values of SICI for each of the No History group and the Diagnosed and Concussion-Like cohorts of the Concussion History group.** The dark lines represent group averages from pre- to post-iTBS. The gray lines represent SICI pre- and post-iTBS for each individual within that cohort. SICI is expressed as a percentage of unconditioned MEP amplitude. Error bars represent the standard error of the mean.

The Group × Time × Sex mixed measure ANOVA for SICI failed to reveal any significant interactions or main effects (Figure 3C). *Post hoc* effect sizes suggest that the absence of significant effects was driven by variability within the History group associated with whether participants reported a diagnosed concussion or a concussion-like event that went unreported/undiagnosed concussion at the time of injury. Both the Diagnosed (Cohen’s *d* = 0.26) and Concussion-Like (Cohen’s *d* = 0.81) cohorts demonstrated enhanced SICI pre-iTBS with a slightly larger effect in the latter. However, both the direction and magnitude of the change in SICI following iTBS was markedly different across the concussion cohorts. The Diagnosed subgroup demonstrated a similar magnitude of facilitation of SICI as the no History group post-iTBS (Diagnosed, Cohen’s *d* = 0.05). In contrast, the Concussion-Like group demonstrated a large suppression of SICI post-iTBS (Concussion-Like, Cohen’s *d* = −1.83; Figure 4).

## Discussion

The current study is the first to investigate the persistent change in motor cortical physiology resulting from concussion during adolescence. We observed baseline increases in motor cortical excitability (MEP amplitude) in a group of individuals who suffered at least one concussion during adolescence. However, baseline metrics of GABA_A_ function (SICI), glutamatergic function (ICF) and RMTs were similar across all individuals, regardless of concussion history. To measure the ability to modulate the mechanisms underlying these networks, we re-assessed MEPs, SICI, ICF and RMT post-iTBS. The group with a concussion history demonstrated less facilitation of MEP amplitude and ICF compared to those without a concussion history. While post-iTBS change in SICI did not differentiate between those with and without a concussion history, we did find evidence that SICI was decreased in individuals whose concussion was not diagnosed at time of injury. In contrast, individuals whose concussion was diagnosed demonstrated the expected increase in SICI post-iTBS. Interestingly, the increase in SICI in the diagnosed group post-iTBS was similar to those with no concussion history.

The primary finding of the present study is that a history of adolescent concussion increased motor cortical excitability but decreased sensitivity to the plasticity inducing protocol iTBS. The greater MEP amplitude for the concussion history group at baseline was unexpected. To our knowledge, this is the first report of changes in motor cortical excitability in asymptomatic individuals. Studies of the chronic effects of concussion in active or retired athletes generally report no baseline differences in MEP amplitude or recruitment curves (for a summary see Table II from Lefebvre et al., 2015). Our observation of increased motor cortical excitability may reflect a neurophysiological interaction between developmental plasticity and concussion neurophysiology that is masked in individuals who continue to play competitive sports as adults. However, the control group in the aforementioned research in collegiate and professional athletes is generally comprised of individuals who played or were playing the same contact sports as the concussed individuals. The majority of our control group were comprised of individuals who participated in competitive sports with minimal risk of head impact (see Table 1 for a list). The reduced exposure to both clinical and subclinical impacts in our no concussion history control group compared to that comprised of non-concussed contact sport athletes may have increased our sensitivity to detect concussion-related effects regardless of the developmental component. Despite sport differences across our control and concussion/concussion-like history groups, differences in overall physical activity are unlikely to explain our results (Ridding and Ziemann, 2010). Of our 15 non-concussed individuals only three individuals did not participate in competitive sport during high school and all our participants played organized sport at some point during elementary and middle school.

MEP amplitude reflects the propensity to recruit excitatory inter-neurons that augment the corticospinal projections to the target muscle (Paulus et al., 2008). The exact mechanisms are uncertain, however the additional interneurons recruited are likely part of more complex oligosynaptic intracortical networks susceptible to complex excitatory and inhibitory interactions and modulation by dopamine and acetylcholine among other neurotransmitters (Ziemann et al., 2015). Magnetic resonance spectroscopy measures of glutamate and GABA show that the typical positive relationship between these neurotransmitters (Stagg et al., 2011; Tremblay et al., 2014a) is absent in individuals up to 3 years post-concussion (Tremblay et al., 2014a). The absence of baseline differences in SICI and ICF between the no history and concussion history groups does not preclude the possibility that the enhanced cortical excitability in the latter is the result of alterations in the balance of glutamate and GABA. The relationship between magnetic resonance spectroscopy measures and TMS derived measures of GABA (SICI, cortical silent period, long-interval cortical inhibition) and glutamate (ICF) is tenuous (Stagg et al., 2011; Tremblay et al., 2014a). However, the absence of baseline differences in SICI and/or ICF also leaves open the possibility that the baseline MEP amplitude difference reflects the effect of specific neuromodulators rather than GABA or glutamate themselves. Pharmacological studies demonstrate MEP amplitude is increased by noradrenergic and serotonergic agonists (Ziemann et al., 2015). Noradrenergic drugs are also known to influence SICI and ICF (Ziemann et al., 2015). In contrast, there is no consensus that serotonergic drugs influence SICI, ICF or other measures like the cortical silent period (Ziemann et al., 2015) leaving open the possibility the baseline differences in MEP amplitude are tied to serotonergic function.

To our knowledge the current study is the first to assess the efficacy of iTBS in asymptomatic individuals with a history of adolescent concussion. iTBS is a form of non-invasive brain stimulation that is thought to be NMDA-receptor dependent (Huang et al., 2007). It is commonly used as a proxy to study experience dependent mechanisms of plasticity involved with increasing synaptic strength, known as LTP. While the effect of iTBS is variable across participants (Hamada et al., 2013), the general trend in cohorts of “healthy individuals” is that iTBS increases MEP amplitude (Huang et al., 2005). The smaller increase in MEP amplitude and ICF we observed post-iTBS in the concussion history group suggests that the injury reduces the ability to modulate intracortical networks by strengthening synapses. Similar decreases in efficacy of LTP-like and long-term depression (LTD) like mechanisms have been reported following both facilitatory and inhibitory protocols of paired associative stimulation in active college football players with a history of multiple concussions (De Beaumont et al., 2012). However, this is the first report of such changes in non-collegiate or non-professional athletes, where participation in competitive sport and concussion history was restricted to adolescence.

The efficacy of iTBS is linked to the propensity to recruit later indirect waves (Hamada et al., 2013). Reduced sensitivity to iTBS in our concussion group is indicative of suppressed late indirect wave networks. However, the pre-iTBS increase in MEP amplitude with a concussion history is difficult to resolve with a simple indirect wave explanation. Instead, the combination of increased MEP amplitudes at baseline and reduced efficacy of iTBS is consistent with a homeostatic metastatic plastic-like mechanism recruited to stabilize neuronal networks and prevent uncontrolled potentiation (Ziemann and Siebner, 2008). The slight reduction in ICF following iTBS for the History group is also consistent with a homeostatic explanation where maintaining high potential for facilitation could also contribute to destabilizing neuronal networks. To our knowledge this is also the first study to assess the effects of plasticity inducing protocols upon SICI and ICF in asymptomatic individuals with a history of concussion.

In contrast to reduced modulation of MEP amplitude and ICF, we did not observe any significant differences in SICI after iTBS. At first glance it would appear that the chronic effects of concussion upon motor cortical plasticity were restricted to modulatory mechanisms within excitatory networks. Both the diagnosed and concussion-like groups revealed similar reductions in the efficacy of iTBS upon MEP amplitude and ICF. In contrast, the effect sizes for SICI were markedly different in both direction and magnitude across concussion cohorts. The diagnosed concussion history cohort demonstrated SICI enhancement post-iTBS that was similar in both direction and magnitude to the no concussion history group whereas those whose injury went undiagnosed/unreported at time of injury demonstrated a large reduction in SICI post-iTBS. Much like MEP amplitude, the reduction in SICI post-iTBS in the concussion-like cohort is consistent with a metaplastic mechanism to stabilize neural networks (Ziemann and Siebner, 2008). Slightly elevated SICI pre-iTBS in the concussion-like cohort may have recruited mechanisms to stabilize neuronal networks given the already elevated efficacy of the networks probed by SICI. The driving force behind the metaplastic response is unknown. The similarity between the concussion cohorts with respect to MEP amplitude and ICF suggest it is not a global change but one to which SICI is particularly sensitive. However, future work in larger cohorts is needed to unequivocally determine whether our diagnosed and concussion like-cohorts represent truly distinct distributions. Within a sample of young healthy adults elevated baseline SICI was associated with greater decreases in SICI post paired associative stimulation, another TMS plasticity inducing protocol (Murase et al., 2015). Therefore, we cannot definitively rule out that our concussion cohorts represent two tails of individual variability within the same distribution that was captured by sampling error given the relatively small diagnosed and concussion-like cohort sizes. However, Murase et al. (2015) also observed that greater baseline SICI predicted greater MEP facilitation post-paired associative stimulation. Yet our cohorts demonstrated consistent reductions in MEP amplitude post-iTBS making such an explanation difficult to completely resolve with the current results. Future work should also focus on the functional significance of the changes in responsiveness to the iTBS protocol. Disinhibition in the form of reduced SICI is correlated to poorer dexterous performance in older adults (Heise et al., 2013). The propensity for disinhibition following iTBS in the concussion-like group raises the possibility of an early indicator of the potential for accelerated loss of dexterous ability (Broglio et al., 2012).

A limitation of the current study is that we did not obtain information regarding medical intervention, time to return to play or other measures of concussion severity to better define the nature of the difference between the diagnosed and unreported/undiagnosed cohorts. We did however, obtain self-report of number of concussions/concussion-like events and duration of symptoms. The diagnosed and unreported concussion-like groups however, reported the same number of concussions/concussion-like events on average. Concussion severity, while linked with increasing GABA_B_ dysfunction (De Beaumont et al., 2007) is also unlikely as duration of symptoms was marginally shorter for the concussion-like history group. One plausible explanation is that the differential effects in SICI may reflect an earlier unrestricted return to sport for individuals who failed to report a concussion-like event or were misdiagnosed compared to those with a medically diagnosed concussion history who presumably would require medical clearance to return to sport. As GABA_B_ dysfunction is known to increase with subsequent concussion (De Beaumont et al., 2007) in professional athletes, it is conceivable that additional head impacts while still recovering from the initial concussion may have the same result. Future work is needed in larger cohorts to definitively identify etiology underlining the diagnosed and undiagnosed demarcation.

Our concussion history group demonstrated slower movement times, but maintained similar reaction times compared to the no concussion history group on a simple response time task. The differences in movement time appear to be most pronounced for our cohort of individuals whose concussion was not diagnosed at the time of the event. The initial difference in movement time between the no history and the concussion history group may reflect subtle differences in the ability to generate smooth accurate movement profiles post-concussion. This is supported by similar findings in postural control (Sosnoff et al., 2011) and implicit motor learning (De Beaumont et al., 2012) long after acute concussion resolution. However, we cannot rule out an alternative compensatory strategy in which increased movement times are a byproduct of incomplete information processing (Broglio et al., 2009; Moore et al., 2014) during the reaction time phase of the task rather than altered movement kinematics/dynamics. Interestingly, movement times were not different in the cued response task. The actual physical movement of the upper limb was identical between the simple and cued response tasks. The only difference was in the information processing stage. Therefore, differences in upper limb kinematics/dynamics, and movement times, should have persisted across task. Instead it appears that similar strategies were used by both the history and no concussion history groups to complete the cued task. Quite possibly the increased information processing required in the cued task may have resulted in incomplete cue processing during the reaction time phase in both groups. iTBS decreased the gap in movement time between concussed group and the non-concussed group in the simple response time task, largely as a result of improved movement times in the concussion-like cohort. None of the physiological measures, either baseline levels or change post-iTBS, could explain the change in movement time on the simple response time task. However, as the change in SICI was specific to a subset of our concussion group we may not be sensitive to the relationship between SICI and behavior given our relatively small samples sizes in the diagnosed (*n* = 9) and concussion-like (*n* = 7) subgroups. We also cannot rule out that the improved movement time may reflect change in a physiological mechanisms not measured. Finally, we also cannot rule out a ceiling effect. Given the no concussion history group already showed significantly faster movement time at baseline they may have been performing at ceiling. As such the differential improvement seen in the concussion history group may have only occurred given their poorer initial performance.

## Conclusion

Overall, the current study demonstrates a chronic change in motor cortical physiology can result from concussion during the adolescent years. In particular, exposure to additional head impacts during the acute injury phase may lead to altered control of inhibitory function in early adulthood, even in the absence of clinical deficits, similar to that seen with advancing age. This raises the possibility that concussion during adolescence may alter the trajectory of maturation. Further research is needed to determine the long-term functional consequences of these early changes in cortical physiology and determine when, if at all, clinical deficits may arise.

## Author Contributions

SKM and SPB conceived and designed the work, SKM, JLM and DNM collected and analyzed the data. SKM, JLM, DNM and SPB all contributed to manuscript preparation.

## Conflict of Interest Statement

The authors declare that the research was conducted in the absence of any commercial or financial relationships that could be construed as a potential conflict of interest.
